# “It went through the roof”: an observation study exploring the rise in PrEP uptake among Zimbabwean female sex workers in response to adaptations during Covid‐19

**DOI:** 10.1002/jia2.25813

**Published:** 2021-10-28

**Authors:** Primrose Matambanadzo, Joanna Busza, Haurovi Mafaune, Lillian Chinyanganya, Fortunate Machingura, Getrude Ncube, Richard Steen, Andrew Phillips, Frances Mary Cowan

**Affiliations:** ^1^ Centre for Sexual Health and HIV/AIDS Research Zimbabwe Harare Zimbabwe; ^2^ Centre for Evaluation London School of Hygiene and Tropical Medicine London UK; ^3^ AIDS and TB Unit Ministry of Health and Child Care Harare Zimbabwe; ^4^ Department of Public Health Erasmus Medical Centre Rotterdam The Netherlands; ^5^ Institute for Global Health University College London London UK; ^6^ Department of International Public Health Liverpool School of Tropical Medicine Liverpool UK

**Keywords:** differentiated care, HIV prevention, PrEP, Sars‐Cov2, sex workers, Zimbabwe

## Abstract

**Introduction:**

*Sisters with a Voice (Sisters)*, a programme providing community‐led differentiated HIV prevention and treatment services, including condoms, HIV testing, pre‐exposure prophylaxis (PrEP) and antiretroviral therapy linkage for sex workers, reached over 26,000 female sex workers (FSW) across Zimbabwe in 2020. Zimbabwe's initial Covid “lockdown” in March 2020 and associated movement restrictions interrupted clinical service provision for 6 weeks, particularly in mobile clinics, triggering the adaptation of services for the Covid‐19 context and a scale up of differentiated service delivery (DSD) models. PrEP service delivery decentralized with shifts from clinical settings towards community/home‐based, peer‐led PrEP services to expand and maintain access. We hypothesize that peer‐led community‐based provision of PrEP services influenced both demand and supply‐side determinants of PrEP uptake. We observed the effect of these adaptations on PrEP uptake among FSW accessing services in *Sisters* in 2020.

**Methods:**

New FSW PrEP initiations throughout 2020 were tracked by analysing routine *Sisters* programme data and comparing it with national PrEP initiation data for 2020. We mapped PrEP uptake among all negative FSW attending services in Sisters alongside Covid‐19 adaptations and shifts in the operating environment throughout 2020: prior to lockdown (January–March 2020), during severe restrictions (April–June 2020), subsequent easing (July–September 2020) and during drug stockouts that followed (October–December 2020).

**Results and discussion:**

PrEP uptake in 2020 occurred at rates <25% (315 initiations or fewer) per month prior to the emergence of Covid‐19. In response to Covid‐19 restrictions, DSD models were scaled up in April 2020, including peer demand creation, community‐based delivery, multi‐month dispensing and the use of virtual platforms for appointment scheduling and post‐PrEP initiation support. Beginning May 2020, PrEP uptake increased monthly, peaking at an initiation rate of 51% (*n* = 1360) in September 2020. Unexpected rise in demand coincided with national commodity shortages between October and December 2020, resulting in restriction of new initiations with sites prioritizing refills.

**Conclusions:**

Despite the impact of Covid‐19 on the Sisters Programme and FSW mobility, DSD adaptations led to a large increase in PrEP initiations compared to pre‐Covid levels demonstrating that a peer‐led, community‐based PrEP service delivery model is effective and can be adopted for long‐term use.

## 1 INTRODUCTION

Much has been written about the negative effects of the Sars‐Cov2 pandemic (Covid‐19) on sex workers’ livelihoods, wellbeing and access to healthcare [1, 2, 3, 4]. Closure of entertainment venues, restrictions on personal mobility and clients’ fears of contracting Covid‐19 have reduced sex workers’ incomes, while strain on health services and disruptions to supply chains decreased their access to healthcare, including HIV testing, prevention and treatment [5]. In some contexts, including Zimbabwe, sex workers have experienced increased stigma against them as potential “disease vectors”, leading to harassment and violence, including from police [6, 7]. There have been calls for policies and programmes to recognize sex workers’ enhanced vulnerability and respond accordingly [3, 8].

The need for ongoing flexibility during this time, however, also provided opportunities for introducing or scaling up existing differentiated service delivery (DSD) models that may previously have been thought too costly or unfeasible to implement. It also refocused attention on the structural drivers of vulnerability [9, 10]. Pre‐exposure prophylaxis (PrEP) may be particularly well suited to testing new DSD approaches, given its recent introduction into national HIV programmes in many sub‐Saharan African countries and initial slow uptake among some populations, including sex workers and adolescent and young women at particularly high risk [11, 12, 13]. Despite successful demonstration projects, PrEP initiation and retention continue to pose challenges to prevention programmes throughout the region, prompting calls for renewed efforts to increase uptake. One method for increasing uptake is by making it more easily available in community settings beyond health facilities [12].

In Zimbabwe, oral PrEP has been offered to sex workers since 2016, when the Ministry of Health and Child Care (MoHCC) adopted World Health Organization (WHO) guidelines. However, widespread access began in 2018 with phased rollout of a 2‐year national Implementation Plan [14]. *Sisters with a Voice (Sisters)* is a nationally scaled, evidence‐based comprehensive HIV prevention and treatment programme for sex workers implemented by the Centre for Sexual Health and HIV/AIDS Research (CeSHHAR) on behalf of the MoHCC and National AIDS Council (NAC) since 2009. *Sisters* reached over 26,000 female sex workers (FSW) yearly across Zimbabwe in 2019 and 2020 with HIV prevention and treatment and sexual and reproductive health services, relying on robust sex worker‐led community mobilization to link sex workers to services, including provision of condoms, lubricants, HIV testing, PrEP and linkage to antiretroviral therapy. The strength of *Sisters* is its integrated model of government ownership, services delivered through a network of fixed‐site and mobile clinics co‐located within MoHCC clinics and sex worker leadership through 370 peer educators supported and supervised by outreach workers [15]. *Sisters* rolled out PrEP in April 2019, screening all HIV‐negative female, male and transgender sex workers attending clinics and drop‐in centres following a pilot introduction as part of a trial [16, 17].

Initially, *Sisters’* PrEP implementation protocol specified that nurses should provide prevention, testing and counselling for all sex workers. All FSW who tested negative were screened for PrEP eligibility, and those who accepted were initiated on PrEP. Newly initiated FSW received 1 month's supply and were encouraged to return to the clinic at any time if they experienced adverse reactions, but otherwise advised to attend monthly follow‐up visits for the first 3 months. After that, FSW were recommended to visit the clinic every 3 months for refills, adherence counselling, HIV testing, as well as checks for sexually transmitted infections and for other sexual and reproductive health services. Following the introduction of Covid‐19 lockdown restrictions in late March 2020, *Sisters* was obliged to close all 10 permanent sites for 1 week and all mobile clinics for 6 weeks, re‐opening them as “essential services” on 6 April 2020 and 18 May 2020, respectively. Subsequently, routine clinic visits were discouraged to “decongest” facilities.

To maintain and expand access to PrEP, services were shifted into the community with greater reliance on peer educators and outreach workers to create demand and provide follow‐up support. This paper describes the Covid‐19‐related DSD adaptations made to the PrEP provision within *Sisters* and explores the effect of these on trends on PrEP uptake.

## 2 METHODS

### 2.1 Adapted approach to provision of PrEP

First, existing peer educators were trained on PrEP by their supervising outreach workers and encouraged to become advocates for PrEP within the sex work communities where they live and work. They disseminated information among their peers, dispelled myths and encouraged increased demand. The sex workers at highest risk, tracked weekly, were prioritized for PrEP discussions and referral to new community “access points” established outside the clinic, comprising agreed meeting points or sex workers’ homes. As shown in Table 1, outreach workers and peer educators joined clinicians to form outreach teams that delivered community‐based PrEP services.

**Table 1:** Adaptations made (and sustained) in response to COVID‐19

	PrEP Screening, initiation and early follow‐up (0‐3 months)			PrEP continuation (+3 months)	
Post Covid‐19	Screening	PrEP initiation visit	Initial follow‐up	PrEP refill	Routine clinical follow‐up
WHEN *Service frequency*	At entry point, first clinic/DIC visit	First visit	One month visit, virtual follow up at 1 week for side effects/adverse events	Every 3 months if tolerating well	Every 3 months. SW receive virtual support for with monthly check ins
WHERE *Service location*	Clinic Drop in centre Community	Clinic Drop in centre Community	Clinic Drop in centre Community/home	Clinic Drop in centre Community/home	Clinic Drop in centre Community/home
WHO *Service provider*	Nurses, outreach teams	Nurses, outreach teams	Nurses, outreach teams	Nurses, outreach teams	Nurses, outreach teams
WHAT *Service package*	Counselling on combination HIV prevention, HIV testing, eligibility screening, adherence counselling	Counselling on combination HIV prevention, Adherence, STI, ARV side effects, eligibility screening	Counselling on combination HIV prevention, Adherence, STI, ARV side effects, HIV Testing	Counselling on combination HIV prevention, Adherence, STI, ARV side effects, HIV testing every 3 months	Counselling on combination prevention, substantial risk screening adherence, assess for signs of acute HIV infections, STI, ARV side effects

ARV, antiretroviral; DIC, drop in centre; PrEP, pre‐exposure prophylaxis; STI, sexually transmitted infection; SW, sex worker.

Second, the use of telehealth was scaled up so that at week 1 and again when due for their 1‐month follow‐up clinic visit, sex workers received follow‐up support from a clinician for side effects and adherence counselling via phone calls. Sex workers could report adverse events via phone and WhatsApp.

Third, ongoing virtual support through peer educators was introduced. We provided mobile data and “talk time” for peer educators, WhatsApp broadcast lists were set up and a communication structure created through which each outreach worker remotely monitored a group of local peer educators, each working with their allocated caseload of sex workers with whom they regularly engaged to address PrEP myths, encourage uptake and adherence, and check concerns. Finally, all PrEP re‐supplies were provided for 3 months at a time, waiving the initial requirement for monthly clinic visits.

This adapted PrEP distribution model remained in place into 2021, providing an opportunity to take stock of its impact and identify lessons for future implementation once commodity supply is restored.

### 2.2 Data collection and analysis

In this study, we included aggregated anonymized individual clinic data for all 19,407 FSW who presented to *Sisters* and tested negative in 2020 and all 6539 FSW initiated on PrEP during 2020. Data were collected from all FSW receiving services within *Sisters* at facility or within the community to whom a unique alphanumeric identifier was assigned based on personal data that can be easily recalled through a series of prompts – mother's first name, FSW's last name, date of birth, sex and district of birth – thus eliminating the necessity of any documentation or clinic card. Data were entered electronically into cloud storage and record synced daily, and as long as a sex worker provided the same information at each visit, her records were linked across services and sites. The data captured included: HIV testing, PrEP screening and initiation, each monthly and then quarterly visit, results from repeat HIV testing, any noted side effects, adverse outcomes and reported adherence. National PrEP initiation data used for comparison are as recorded in the DHIS2 system by MoHCC. Ethical approval for this study was granted as part of the wider AMETHIST Consortium group of studies by the Medical Research Council of Zimbabwe (MRCZ/A/2559) and Liverpool School of Tropical Medicine (ref:19‐115RS).

## 3 RESULTS AND DISCUSSION

In total, 19,407 HIV‐negative individual sex workers were screened for PrEP of whom 33.7% (*n* = 6539) accepted PrEP. Initiations were highest among sex workers aged 20–24 years at 33% (2152/6539), followed by 21% among those aged 25–29 (1382/6539). Lowest uptake was among FSW who were 40 years old or over, at 5% among the 40‐ to 44‐year olds and under 2% for those 45 years and above. PrEP continuation data were only available for 5653 FSW (data for 10 out of 61 sites were unavailable). Retention at 1 month was 40% (*n* = 2269), 27% (*n* = 1509) at 3 months and 14% (*n* = 803) at 6 months.

Following Covid‐19‐related closures and amid continuing movement restrictions, adaptations to PrEP provision were introduced as facilities reopened, rapidly scaling up existing DSD models that shifted many routine functions from clinics to the community. 2020 PrEP uptake within *Sisters* ranged between 212 and 315 initiations per month until the emergence of Covid‐19 in Zimbabwe. Beginning around May 2020, the uptake of PrEP consistently increased monthly, until it reached 1360 initiations in September 2020. Unfortunately, the unexpected rise in demand coincided with national commodity shortages from October to December 2020 due to delayed shipments. This stock rupture necessitated restrictions on new initiations with higher volume sites halting initiation altogether. Overall, we saw 746 PrEP initiations January–March 2020, prior to Covid‐19, 1161 between April and June 2020 with most intense restrictions, 3084 following easing of restrictions from July to September 2020 and 1548 during October–December 2020 when drug stockouts were experienced. Despite the stock rupture, seroconversion was reported in 2/6539 FSW initiated on PrEP within *Sisters* in 2020. Low rates of seroconversion despite stockouts may have been due to prioritization of refills over new initiations between October and December 2020.

**Figure 1:**
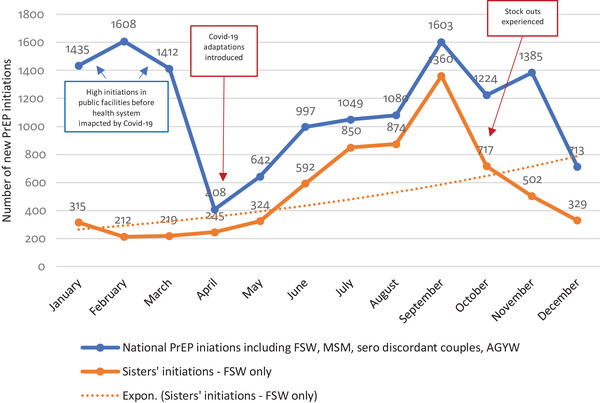
National and Sisters only PrEP initiations January−December 2020. AGYW, adolescents girls and young women; FSW, female sex workers; MSM, men who have sex with men; PrEP, pre‐exposure prophylaxis.

Figure 1 demonstrates the steep rise in PrEP uptake within *Sisters* following these adaptations.

Figure 1 also shows *Sisters’* increased contribution (63%) to national PrEP initiations between April and December 2020, compared with a contribution of 16% between January and March 2020 prior to adaptations within *Sisters*. Prior to the Covid‐19 pandemic, PrEP initiations within the public sector and among other MoHCC implementing partners were four to five times higher than those within *Sisters*, suggesting DSD adaptations made within *Sisters* may have facilitated rapid recovery from the impact of Covid‐19 not witnessed in settings where similar shifts to peer‐led community‐based delivery of PrEP services were not immediately possible.

**Figure 2:**
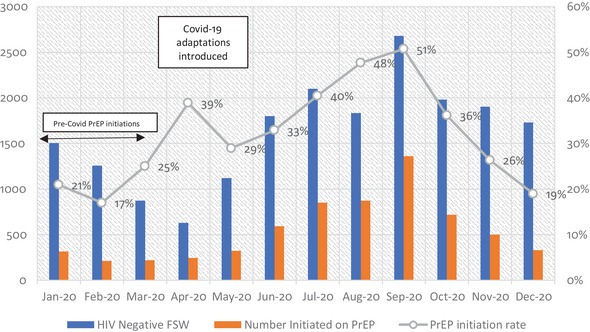
Sisters PrEP initiation rate January−December 2020. FSW, female sex workers; PrEP, pre‐exposure prophylaxis.

FSW reported that the Covid‐19 pandemic reduced their ability to procure clients due to a combination of factors, such as closure of bars, restaurants, truck stops and other sex work venues; restrictions on personal mobility; and clients’ fears about contracting Covid‐19 from sex workers. Anecdotal evidence suggests that a shortage of clients increased competition between sex workers, reducing their ability to negotiate condom use and thus heightening their concerns about HIV risk. Greater risk perception may have, in turn, increased sex workers’ openness to PrEP as an alternative prevention strategy resulting in steady increase in PrEP initiation rates among negative FSW to a peak of 51% in September 2020 before stockouts were experienced as shown in Figure 2. Another factor likely to have contributed to interest is sex workers’ reduced movement around the country. As transport hubs and borders were closed, with inter‐ and intra‐city travel restricted, sex workers were unable to migrate out of Zimbabwe or move between locations in search of work.

## 4 CONCLUSIONS

Globally, Covid‐19 has disrupted healthcare across regions, including negatively affecting many HIV programmes and reducing access for vulnerable populations, such as sex workers [1, 5]. In response, a range of adjustments to service delivery models are being introduced, offering an unexpected real‐time experiment in DSD. Covid‐19‐related service disruptions to Zimbabwe's sex work programme services led to adaptations introduced in April 2020 and sustained throughout the year to scale‐up community‐based PrEP service delivery, significantly boosting PrEP uptake among FSW within the national context. The experience of *Sisters* in Zimbabwe provides one example of how DSD adaptations to PrEP distribution protocols can lead to a rapid increase in its uptake among FSW. A peer‐led, community‐based PrEP service delivery model is effective and can be adopted for long‐term use.

## COMPETING INTERESTS

The authors declare that they have no competing interests.

## AUTHORS’ CONTRIBUTIONS

PM led *Sisters’* Covid‐19‐related adaptations and conceived the idea for the paper. JB wrote the initial draft and coordinated contributions from other authors. HM and LC led analysis of programme data. FM and NG provided comments on early drafts. RS, AP and FC contributed to interpreting the data and re‐writing text. All authors have read and approved the final version of the manuscript.

## FUNDING

The Global Fund to Fight AIDS, TB and Malaria (GFATM), PEPFAR, USAID and the Elton John AIDS Foundation.
